# Neurophysiological and Behavioral Responses of Mandarin Lexical Tone Processing

**DOI:** 10.3389/fnins.2017.00095

**Published:** 2017-03-06

**Authors:** Yan H. Yu, Valerie L. Shafer, Elyse S. Sussman

**Affiliations:** ^1^Department of Communication Sciences and Disorders, St. John's UniversityQueens, NY, USA; ^2^Ph.D. Program of Speech-Language-Hearing Science, The Graduate Center, City University of New YorkNew York, NY, USA; ^3^Dominick P. Purpura Department of Neuroscience, Rose F. Kennedy Center, Albert Einstein College of MedicineNew York, NY, USA

**Keywords:** mismatch negativity, Mandarin lexical tone, interstimulus interval, late negativity, cross-language speech processing, sensory memory, event-related brain potential

## Abstract

Language experience enhances discrimination of speech contrasts at a behavioral- perceptual level, as well as at a pre-attentive level, as indexed by event-related potential (ERP) mismatch negativity (MMN) responses. The enhanced sensitivity could be the result of changes in acoustic resolution and/or long-term memory representations of the relevant information in the auditory cortex. To examine these possibilities, we used a short (ca. 600 ms) vs. long (ca. 2,600 ms) interstimulus interval (ISI) in a passive, oddball discrimination task while obtaining ERPs. These ISI differences were used to test whether cross-linguistic differences in processing Mandarin lexical tone are a function of differences in acoustic resolution and/or differences in long-term memory representations. Bisyllabic nonword tokens that differed in lexical tone categories were presented using a passive listening multiple oddball paradigm. Behavioral discrimination and identification data were also collected. The ERP results revealed robust MMNs to both easy and difficult lexical tone differences for both groups at short ISIs. At long ISIs, there was either no change or an enhanced MMN amplitude for the Mandarin group, but reduced MMN amplitude for the English group. In addition, the Mandarin listeners showed a larger late negativity (LN) discriminative response than the English listeners for lexical tone contrasts in the long ISI condition. Mandarin speakers outperformed English speakers in the behavioral tasks, especially under the long ISI conditions with the more similar lexical tone pair. These results suggest that the acoustic correlates of lexical tone are fairly robust and easily discriminated at short ISIs, when the auditory sensory memory trace is strong. At longer ISIs beyond 2.5 s language-specific experience is necessary for robust discrimination.

## Introduction

### Mandarin lexical tone

“Lexical tone” is a linguistic term that describes language-specific use of pitch patterns to distinguish lexical meaning. Pitch is the perception of changes in the physical (acoustic) property of fundamental frequency (F0). The F0 patterns of lexical tone reflect the rate of vocal fold vibration during the production of a sound (Yip, 2002). A language is considered a tone language if a conventional change in the pitch pattern of a word results in a change in meaning of that word (Yip, 2002, p.1). All languages use segmental changes to contrast meaning (e.g., English consonants /r/ to /l/ in “rust” vs. “lust,” or vowels /I/ in “hit” vs. /æ/ “hat”). A tone change is phonemic when the change of this one property leads to a meaning change. The current study assesses how native speakers of a non-tone language perceive and process lexical tone.

**Figure 1:**
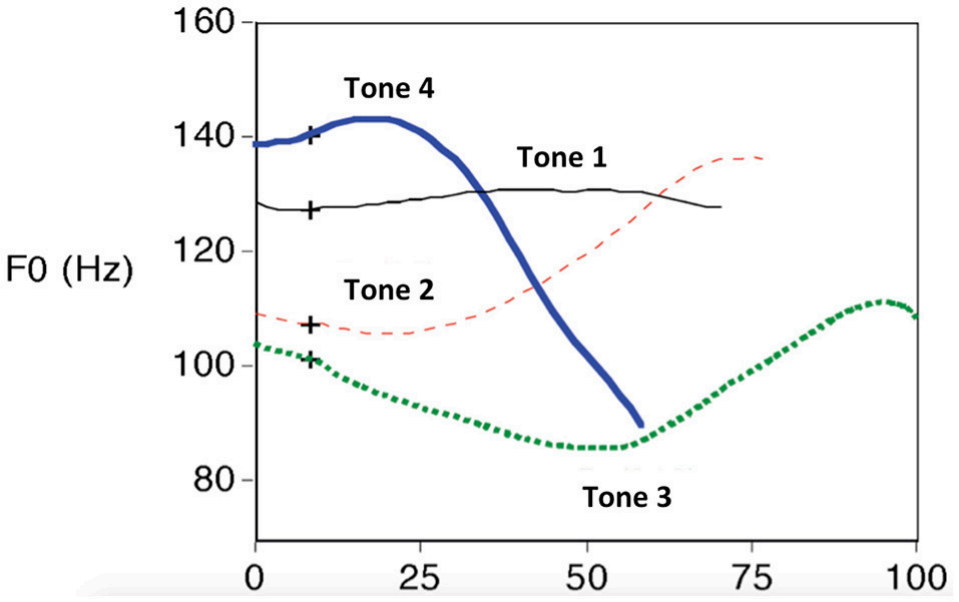
**Mandarin tone produced in isolation**. Modified from Xu (1997).

Mandarin is a tone language, which has one level tone and three contour tones (in stressed syllables). In isolated syllables, Tone 1 (T1, e.g., bi1,“逼,” “to force”) has a high and essentially level F0 contour. Tone 2 (T2, e.g., bi2,“鼻” “nose”) has a dipping start and then changes into a rising F0 contour approximately 20% of the way into the duration of the vowel. Tone 3 (T3, e.g., bi3, “笔” “pen” or “比” “to compare”) also has a dipping start and then changes into a rising F0 contour at a point approximately 50% of the duration of the syllable; and Tone 4 (T4, e.g., bi4,“壁,” “wall”) has a falling F0 contour (Howie, 1976). Native speakers of Mandarin make use of these tone patterns to rapidly access lexical meaning. In contrast, non-native listeners who do not speak a tone language show poor perception (discrimination and identification) (Gandour and Harshman, 1978; Xu et al., 2006) and late second language (L2) learners of a tone language often access the incorrect lexical representation due to poor lexical tone perception (Kaan et al., 2008). Figure 1 modified from Xu (1997) shows the lexical tone contour for monosyllabic Mandarin word in isolation (for more information, see Shen, 1990; Xu, 1999; Chen, 2000; Hua and Dodd, 2000).

### Interstimulus interval and three processing modes in the behavioral literature

Speech discrimination in a non-native language is generally challenging because non-native listeners often do not have phonological categories from the first language that match with those of the non-native language. In some cases, two speech sounds from a non-native language are assimilated into the same category of the listener's first language, leading to difficulty in categorizing and discriminating these speech sounds (Best and Tyler, 2007; Strange, 2011). In this case, to succeed at discrimination, non-native listeners must rely on acoustic differences between the speech sounds (e.g., van Wijngaarden et al., 2002; Strange, 2011).

The sensory trace of an acoustic signal decays over time, and thus, discrimination of two non-native contrasts for which a speaker lacks two distinct phonological categories will suffer with increasing time delays. A few studies have shown that increased interstimulus interval (ISI) between two non-native speech sounds that assimilate into the same phonological category of the first language of a listener results in poorer discrimination and categorization of these speech sounds (Pisoni, 1973; Werker and Logan, 1985). For example, at very brief ISIs (less than 500 ms), American English listeners could discriminate dental [d] vs. retroflex [d] (which is phonemic in Hindi, but not English) (Werker and Logan, 1985; also see Shafer et al., 2004). However, at a longer ISI of 1,500 ms, the American English listeners no longer showed good discrimination and categorization of this Hindi speech sound pair. The authors suggested that the American English listeners relied on their native phoneme categories and assimilated the dental and retroflexed speech sounds into the same American English phoneme category /d/. These few behavioral studies showed that when the ISI is very short (e.g., less than approximately 500 ms), the acoustic/phonetic information (or code) is available and discrimination can be good for within-category non-native contrasts. Acoustic (and possibly phonetic) representations of speech are maintained in echoic sensory memory (Pisoni, 1973; Werker and Logan, 1985; Burnham et al., 1996), and thus decay rapidly. When the ISI is long (e.g., lengthened to greater than 1,500 ms), the acoustic/phonetic information has decayed. These findings have been interpreted as indicating that listeners can perform these tasks using three different processing modes (or codes), in which acoustic and phonetic modes can be used under short ISI conditions, while only the phonemic mode is available under long ISI conditions (Werker and Logan, 1985). In other words, both language-universal representations at the phonetic level and language-specific representations at the phonological level of speech distinctions co-exist. However, the phonetic information is not represented in long-term memory, and thus the memory trace decays over time.

As pointed out by Phillips (2001), much has been reported about the analog representation of the acoustics of speech at the peripheral auditory level that is independent of language experience and the discrete, abstract phonological representation that is shaped by language experience. But less understood is the phonetic level of processing. For example, on the one hand, the majority of behavioral and neurophysiological evidence supports greater sensitivity to between-category than within-category phonological contrasts as indicated by better behavioral performance and larger brain responses; on the other hand, some studies also have reported similar neurophysiological responses to between-category (native) and within-category (non-native) speech contrast (e.g., Rivera-Gaxiola et al., 2000a,b). Similar to early findings on behavioral speech perception, Phillips (2001) attributes this mixed evidence to the different types of phonetic categories (e.g., vowels vs. consonants). Vowel perception is more continuous, and consonant perception is more categorical. It is, however, unclear where lexical tone perception falls on this spectrum. We propose that language experience will modulate the strength of lexical tone representation and the rate of sensory memory decay. More specifically, phonological representations in long-term memory can refresh sensory memory. Strong sensory representations can only be retained for language-specific lexical tone under the long ISI condition. The alternative hypothesis, however, is that the acoustic distinctiveness for lexical tone pairs is fairly robust even for non-native listeners; In this case, sensory memory of lexical tone will be less influenced by language experience, and similar patterns will be observed behaviorally and neurophysiologically for both the native Mandarin and the monolingual English listeners under both ISI conditions. One goal of this paper is to address which of these two hypotheses has better support.

Studies that have examined the account of three processing modes (acoustic, phonetic and phonemic modes) for speech sounds focused on consonant and vowel contrasts. Lexical tone differs from consonants and vowels in that tone can be viewed as a non-segmental feature superimposed mostly on the vowel, but also as a segmental feature given its functional role of distinguishing meaning (Burnham, 1986). It is less clear whether lexical tone will show the same pattern of processing as found for vowels and consonants. As an example, the one study undertaken with tone, a Thai lexical-tone training study, revealed no effect of ISI on perception (Wayland and Guion, 2004). In this study, Wayland and Guion examined native English and native Chinese listeners' ability to identify and discriminate mid- vs. low-tone Thai contrasts before and after auditory training using short and long ISI presentation rates (500 vs. 1,500 ms). They found no within-language group effects of ISI in any of the three language groups (Native Thai, native English or native Chinese). These results challenge the account of three processing modes because based upon this account we should expect lower performance under the long ISI condition than the short ISI condition in the English speakers and the Chinese speakers, especially before training (Pisoni, 1973; Werker and Logan, 1985; Burnham et al., 1996). However, two reasons for this lack of ISI effect in Wayland and Guion (2004) are that lexical tone contrasts are acoustically more salient than consonant contrasts, and/or that the ISI of 1,500 ms was not long enough to affect acoustic processing.

### Neurophysiological measures of speech processing

Speech discrimination tasks used in behavioral studies require immediate and overt responses from subjects, thus the result is affected by attention, inhibition, motivation, decision-making, motor dexterity and other cognitive factors. The language-specific lexical and sublexical features of the stimuli also affect the results (e.g., Hulme et al., 1991). Consequently, these behavioral methods alone cannot easily tease apart the contributions from different cognitive and linguistic factors. Neurophysiological measures of discrimination are an ideal method to examine the processes that underlie speech discrimination and to compare processing of native and non-native speech sounds. In particular, the passive-listening mismatch negativity (MMN), event-related brain potential (ERP) offers an excellent method for studying the effects of ISI on speech sound processing (Näätänen et al., 1978, 1987; Mäntysalo and Näätänen, 1987; Böttcher-Gandor and Ullsperger, 1992; Sams et al., 1993). Lengthening the ISI between two different tones can be used to examine the duration of sensory memory because longer ISIs lead to greater sensory memory trace decay for a stimulus, and, therefore, reduced MMN amplitude (Böttcher-Gandor and Ullsperger, 1992; Sams et al., 1993; Winkler et al., 2001). Thus, manipulating the ISI between stimuli allows an estimate of the short-term sensory memory duration for the standard stimulus (Mäntysalo and Näätänen, 1987; Näätänen et al., 1987; Böttcher-Gandor and Ullsperger, 1992; Sams et al., 1993). Most ERP studies manipulating ISI have used auditory tones as stimuli. In adults, MMN can be elicited with an ISI as long as 10 s when the stimuli are auditory tones that differ in frequency by 10 percent (e.g., Standard: 1,000 Hz, Deviant: 1,100 Hz in Sams et al., 1993). However, it is unclear to what extent such results can be generalized to speech processing. For example, Ceponiene and colleagues found that when the stimuli were auditory tones (1,000 & 1,100 Hz), there was no MMN amplitude difference between the children with high and low nonword repetition (NWR) performance under either 350 ms or 2,000 ms ISI condition. However, when the stimuli were speech (/baka/ & /baga/), MMNs were obtained only in the high performers albeit the MMN was reduced in amplitude in the long ISI condition.

To date, manipulating ISI has not been used as a neurophysiological method to examine how Mandarin tone is represented in sensory memory. The current study is designed to address such a gap in the literature.

### Mismatch negativity: cross-language lexical tone evidence

Cross-linguistic studies have shown that the MMN measure reflects experience with speech (for consonants, e.g., Dehaene-Lambertz, 1997; Sharma and Dorman, 1999, 2000; Shafer et al., 2004; for vowels, e.g., Näätänen et al., 1997; Szymanski et al., 1999; Winkler et al., 1999a,b; Hisagi et al., 2010) and that this finding extends to phonemic tone contrasts. For example, the amplitude of MMN is larger for between- than within-category F0 differences in native Mandarin listeners (Xi et al., 2010; Yu et al., 2014). Ren and colleagues found that MMN was larger when pitch was used phonetically than when it served a prosodic (intonation) function (Ren et al., 2009). The amplitude of MMN has also been linked to the acoustic distance of lexical tone contrast (Chandrasekaran et al., 2007b,a; Lee et al., 2012; Yu et al., 2014). Xi et al. (2010) found that both within- and between-category tonal deviants generate MMNs in native Mandarin listeners, with larger MMNs elicited from the between-category contrast. The above-mentioned studies have provided important knowledge about the general effects of language experience on the MMN responses. However, the short ISI and the fairly simple, monosyllabic stimuli allow for discrimination based primarily on acoustic information. Specifically, the more robust MMN across the phonemic boundary could primarily be an enhanced response to the acoustic properties of the stimuli. A study using more complex stimuli with longer ISIs would allow a clearer view of how long-term memory representations of natural speech are instantiated at the cortical level.

Another important question that remains unclear from previous neurophysiological research is whether and to what degree non-native listeners are sensitive to acoustic distinctiveness of lexical tones. Some studies found no evidence of neural sensitivity to the degree of dissimilarity of lexical tone contrasts. For example, Chandrasekaran et al. (2007b) compared MMN responses using an “easy” contrast (an acoustically more distinct pair: Mandarin Tone 3 vs. Tone 1) and a “hard” contrast (an acoustically less distinct pair: Mandarin Tone 3 vs. Tone 2) and observed no difference in the MMN amplitude between the T1-T3 and T2-T3 conditions for the English listeners, whereas Mandarin listeners showed a larger MMN for the easy than the hard contrast. This lack of neural sensitivity as measured by MMN to the degree of acoustic dissimilarity in lexical tone contrasts appears to contradict the behavioral literature, in which better performance is observed in non-native listeners for acoustically-more-distinct contrasts (e.g., T1-T3) than for acoustically-less-distinct contrasts (e.g., T2-T3) (Gandour and Harshman, 1978; Gottfried and Suiter, 1997; So and Best, 2010). It is possible that the acoustic differences between the standard and deviant stimuli were sufficiently salient even for the “hard” contrast (T3-T2) to allow non-native listeners to exhibit large-amplitude MMNs. However, this does not explain why experience with the lexical tone contrasts only enhanced the easy contrast for native listeners. Further investigation will be necessary to understand the neural mechanism of lexical tone processing in non-native speakers of tonal language via using a paradigm that is more likely to engage processing at the phonological processing.

### The influence of stimulus complexity and within-category variability on phonemic processing in the MMN paradigm

According to Sussman (2007), the principal factor that governs the MMN response is the standard formation context. Phonology involves abstract mental representation. A paradigm that is likely to result in the listener engaging phonemic abstraction is one in which the speech stimuli include within-category variation (Politzer-Ahles et al., 2016). Multiple tokens of the deviant and standard stimuli provide this within category variation. MMN responses from a high token-variability paradigm can lead to a different pattern of results than found from a paradigm in which only one token of standard and one of a deviant stimulus are used, because the single token paradigm allows for discrimination solely on the basis of acoustic difference (Hestvik and Durvasula, 2016). In addition, increased phonological complexity of stimuli is likely to lead to greater reliance on the phonological level of processing. Previous studies have demonstrated that stimulus complexity plays a role in determining whether naïve listeners can quickly access the phonetic details of vowel production (Strange et al., 2005, 2009; Strange, 2011). In addition, an increase in stimulus within-category variability and stimulus complexity are essential ways to generate ecologically more valid, speech perception tasks (Strange, 2011). Only a few MMN studies have used bisyllabic or multisyllabic non-words as stimuli (e.g., “/ebuzo/” vs. “/ebuzo/”used in Dehaene-Lambertz et al., 2000; “tado” vs. “taado” used in Hisagi et al., 2010; “Sicherheit” vs. “Sauberkeit” in Hanna and Pulvermüller, 2014; “tatata” vs. nonspeech counterpart in Sussman et al., 2004). The role of speech token variability on MMN responses and ecological validity have seldom been discussed, and almost all the Mandarin tone studies have used simple single-token single vowel (e.g., “yi” is used in several studies) or monosyllabic consonant-vowel stimuli (“pa” in Xi et al., 2010; Yu et al., 2014; “tu” in Lu et al., 2015). Considering that compared to consonant contrasts, the acoustic distinctions for lexical tone contrasts are relatively robust with fundamental frequency unfolding over the entire syllable, it is important to take stimulus complexity and the experimental paradigm into consideration. In the current study, to increase the likelihood that participants engaged phonological processing, we used multi-token within-category stimuli for each Mandarin tone category.

### Late negativity

A late negativity (LN) observed at frontal sites and often following the MMN has been reported in an increasing number of studies (Čeponiene et al., 1998; Korpilahti et al., 2001; Shestakova et al., 2003; Hill et al., 2004; Shafer et al., 2005; Kaan et al., 2007; Bishop et al., 2010; Datta et al., 2010; Ortiz-Mantilla et al., 2010). The LN serves as an additional index that discrimination has occurred and there is some evidence that the LN will be seen in the absence of the MMN in listeners with weak phonological skills, such as language impairment (Shafer et al., 2005; Barry et al., 2009; Bishop et al., 2010), and possibly non-native listeners (Kaan et al., 2007, 2008). Kaan et al. (2007); Kaan et al., 2008 conducted a Thai lexical tone training study using two Thai tone contrast pairs (low-falling vs. mid-level tone; mid-level vs. high-rising tone) and found a language group effect for an LN. A left lateralized LN was observed for the high-rising deviant condition for the English and Chinese groups post training. However, It is possible that this LN was actually an MMN to the mid-high tone contrast because the high-rising and mid-level tones do not diverge significantly in F0 until 300 ms later than for the low-falling compared to midlevel tone. In Mandarin, T2 and T3 have very close onset F0, and do not diverge significantly until 20% into the syllable. Native listeners rely primarily on the F0 contour while non-native listeners rely mostly on the F0 onset, offset or the average F0 for behavioral discrimination (Gandour and Harshman, 1978). Therefore, it is important to also examine whether both an MMN and LN are elicited in lexical tone discrimination and how the timing of these components relates to time of the tone stimulus difference.

### The present study

In the present study, we used an MMN design to examine Mandarin lexical tone processing in native and non-native listeners under two different memory-delay conditions (short and long ISIs). Previous MMN studies on the neural plasticity of lexical tone in non-native speakers (e.g., Chandrasekaran et al. (2007a) and Kaan et al. (2008), have used only short ISI conditions that allow for discrimination on the basis of acoustic-phonetic cues but may preclude discrimination of longer-term memory content that accesses lexical information. In this study, we are extending the current literature of cross-language lexical tone processing by comparing neural responses under short and long ISI conditions. We predicted that when the ISI was short, both English and Mandarin listeners would be able to rely on acoustic-phonetic cues for discriminating the lexical tone contrasts, whereas when the ISI was long, the acoustic cues would be degraded. In this latter case, both English and Mandarin listeners were expected to have to make use of long-term memory traces of native phonology to update the sensory memory trace. This would result in a language group difference in the MMN amplitude only for the long ISI condition. A second possibility is that both English and Mandarin listeners have strong acoustic-phonetic representations for lexical tone. In this case, there will be no group differences at either ISI condition. A third possibility is that Mandarin listeners will have larger MMN amplitudes than American English listeners at both ISIs if the native-language phonological representations somehow sharpen the initial memory trace. In this third case, we expect Mandarin listeners have larger MMN amplitudes than the English listeners at both the short and long ISI condition. Less is known about the LN in relation to cross-linguistic processing, so as a working hypothesis, we predict that LN, if present, will show a similar pattern to the MMN.

## Methods

### Participants

This study recruited a total of 68 participants. Data from 31 monolingual adult native English speakers (16 participants in the short ISI condition, and 15 participants in the long ISI condition, age range: 20–42 years) with no exposure to tone languages and 32 adult native Mandarin speakers (16 participants in each ISI condition, age range: 21–40 years) were included in the analysis (See Table 1). All Mandarin participants were born in Mainland China, and had to have completed at least 12 years of formal education in China. Some participants could speak another dialect of Chinese, but all participants reported on the language questionnaire that Mandarin was their only or most often used language prior to coming to the United States. A total of five participants were excluded due to incomplete participation, or excessive noise in the EEG. All participants passed a hearing screening and had no history of neurological impairment. Participants had no formal music training in the prior 10 years, and did not play any instruments on a regular basis (Alexander et al., 2005; Wong et al., 2007). The two language groups were closely matched with respect to age and years of formal education. The handedness questionnaire adapted from the Edinburgh handedness inventory (Oldfield, 1971) by Cohen (2008) was administered to all the participants (Table 1). The participants were paid $10 per hour for their voluntary participation. Voluntary informed consents were obtained from all the participants at the beginning of their participation in the study. The study was approved by the human subject research institutional review board at the Graduate Center, City University of New York, and was conducted in compliance with the *Declaration of Helsinki*.

**Table 1:** **Participants**.

**Participant group**	**Age (range, *SD*)**	***N* (gender)**	**Handedness**
English Long ISI	28.4 (20–42, 6.6)	15(8M, 7F)	1 LH,14 RH
English Short ISI	29.8 (22–41, 5.6)	16(7M, 9F)	1 LH, 15 RH
Mandarin Long ISI	29.1 (23–40, 5.3)	16(9M, 7F)	all RH
Mandarin Short ISI	25.9 (21–36, 4.5)	16(8M, 8F)	1 ambidextrous, 15 RH

*Age, gender, and handedness information of the four groups of participants (2 interstimulus interval conditions by 2 language background conditions)*.

### Stimuli

Natural speech sounds containing both phonetically relevant and phonetically irrelevant acoustic variations were produced by a female native speaker of Mandarin, and digitized at a sampling rate of 22,050 Hz. The stimuli consisted of three nonsense bisyllabic word types (/gupa/, /gipa/, and /gypa/) with three tone variations (Tone 1/T1, Tone 2/T2, and Tone 3/T3) on the first syllable only; the second syllable was always “pa” with T1. The final set of 11 stimuli consisted of two tokens for the T1 deviant /gu1pa/, two tokens for the T2 deviant /gu2pa/, three tokens for the standard T3 /gu3pa/, two tokens of standard T3 /gi3pa/ and two tokens of standard /gy3pa/. These eleven tokens were selected from a larger set of recordings that were piloted extensively. /gi3pa/ and /gy3pa/ were not included in the current ERP analysis because these tokens have a dual function serving as tone standard and vowel deviant stimuli concurrently. Including these vowel variants further increased the ecological validity of the stimuli by incorporating greater variability.

**Figure 2:**
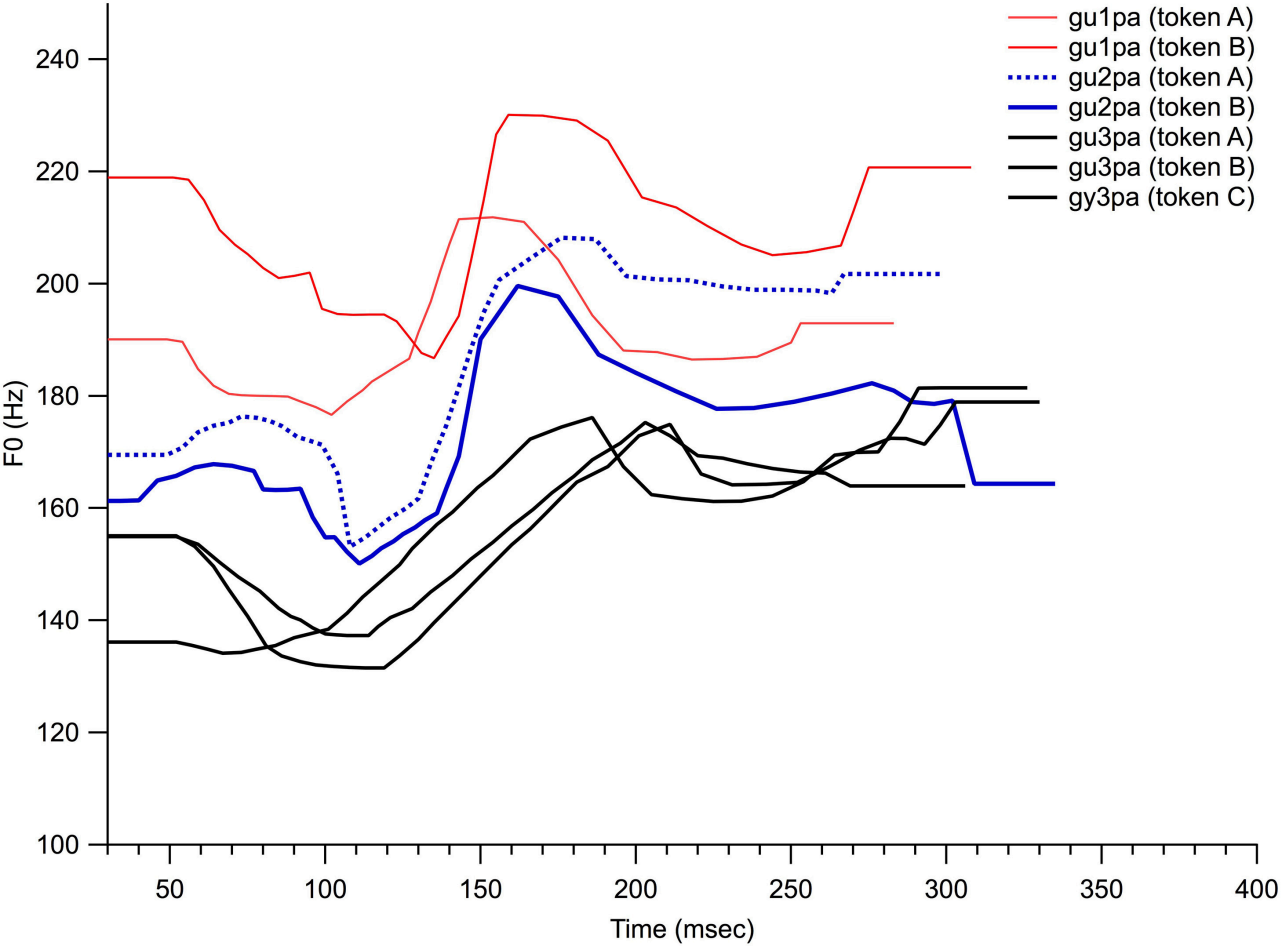
**The lexical tone (F0) contour of the stimuli**. Two tokens of tone 1 (gu1pa), two tokens of tone 2 (gu2pa) and three tokens of tone 3 (gu3pa).

Table 2 displays acoustic measurements of the stimuli used in this study, and Figure 2 displays the fundamental frequency (F0) contour of the /gu?pa/ stimuli used in this study. During the pilot period of the study, six native speakers of Mandarin (three of them are doctoral students majoring in the speech and language sciences) listened to the stimuli and reported that the prominent difference among these stimuli was the intended lexical tone difference. Phonetically irrelevant acoustic differences (e.g., overall amplitude, overall duration and voice onset time of the stop consonants among others) were equivalently distributed across each tone category (measurements made in Praat 4.1 and Sound Forge, version 8). The average duration of the stimuli was 331 ms (range: 291–355 ms, *SD* = 19.7), and the average intensity of the stimuli was 70 dB SPL (range: 67–73, *SD* = 1.9 dB SPL).

**Table 2:** **The acoustic measures of the stimuli**.

**Stimuli**	**gu1pa**		**gu2pa**		**gu3pa**		
	**Token 1**	**Token 2**	**Token 1**	**Token 2**	**Token 1**	**Token 2**	**Token 3**
F0-gu (Hz)	186	214	174	166	140	142	143
F0-pa (Hz)	194	214	202	184	167	168	171
F0 onset:gu (Hz)	190	219	169	161	155	155	155
F0 offset:gu (Hz)	182	207	175	167	142	136	141
Duration:overall (ms)	291	343	312	351	320	326	346
Duration:gu (ms)	115	124	115	119	132	139	134
Duration: pa (ms)	175	199	197	232	188	187	212
Intensity:overall (dB)	71.7	72.1	70.8	72.5	68.8	70.9	70.8
Intensity:gu (dB)	69.3	71	72.5	73.6	69.3	70.6	71.6
Intensity:pa (dB)	73	72.6	70	72.1	68.4	71.1	70.4
**FORMANT FREQUENCY**							
F1:gu (Hz)	340	364	339	349	345	345	341
F2:gu (Hz)	1158	1300	1247	1194	1013	1102	1154
F3:gu (Hz)	2794	2902	2799	2758	2622	2630	2688
F1:pa (Hz)	653	638	744	664	722	766	778
F2:pa (Hz)	1394	1487	1509	1472	1538	1465	1478
F3:pa (Hz)	2607	2697	2760	2737	2681	2648	2730

*“gu” stands for the first syllable, and “pa” stands for the second syllable of the stimuli. There are two tokens of tone 1 (gu1pa), two tokens of tone 2 (gu2pa) and three tokens of tone 3 (gu3pa). Four more tokens of tone 3 (two tokens of gy3pa and two tokens of gi3pa) were used in the experiment, and the acoustic measures of these four tokens are included in the appendix*.

### Procedure

During the ERP and behavioral experiment, participants were seated in a sound- and electrically-shielded booth. Stimulus presentation and response collection were implemented using E-Prime software (Schneider et al., 2002). The stimuli were presented free field over two loudspeakers, one meter in front of and 1 m behind and above the listener at 72 dB SPL. The total duration of the experiment lasted approximately 3 h, including preparation and break times.

### ERP experiments

**Figure 3:**
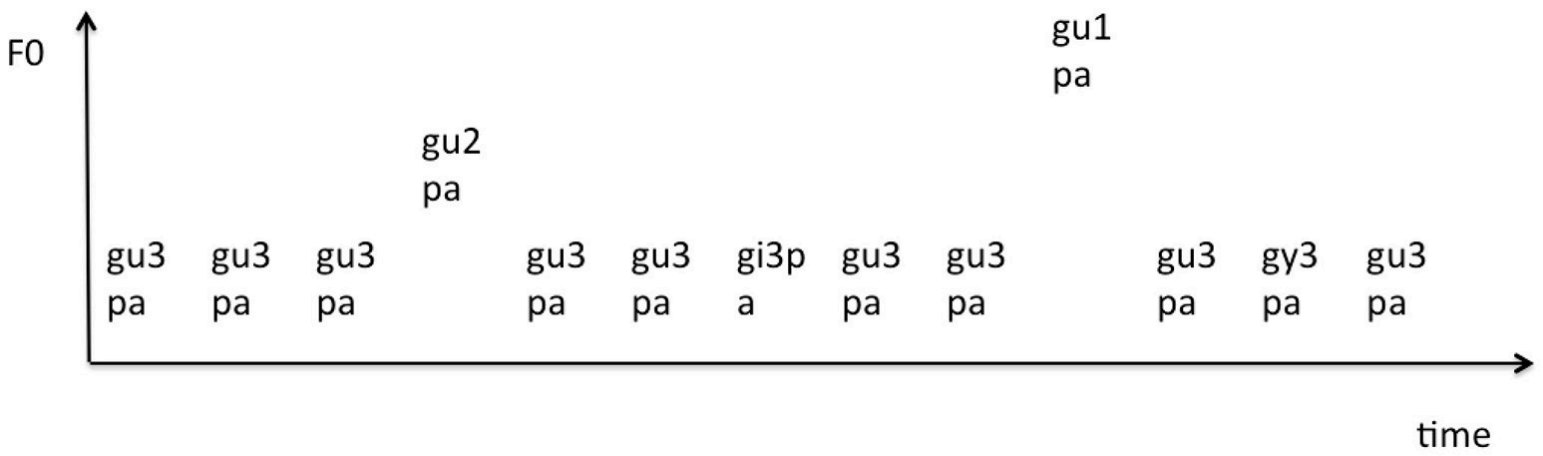
**Schematic of the ERP experiment (Standard condition, gu3pa, 1,280 trials, 62.2%; Tone deviants: gu1pa & gu2pa, 200 trials each, 9.7% each)**. A stimulus onset asynchrony (SOA) of 900 ms for the short interstimulus interval (ISI) condition and 3,000 ms for the long ISI condition were used. Note: gi3pa and gy3pa are also part of standard tone condition, but not included in the analysis.

A passive oddball paradigm was used in which attention was directed toward watching a movie with the sound muted. Twenty blocks with 103 stimuli in each block were presented with an inter-block interval of 20 s. The standard trials consisted of three syllable types (gupa, gipa, gypa) in which the first syllable was Tone 3 (gu3, gi3, gy3) and had the following percentages: three tokens of /gu3pa/ occurred on 62.2%, two tokens of /gy3pa/ on 9.7%, and two tokens of /gi3pa/ on 9.7% of the trials. The tone deviants were two tokens of Tone 1 /gu1pa/ on 9.7 % of the trials and two tokens of Tone 2 /gu2pa/ on 9.7% of the trials. A total of 200 deviant trials were delivered per category (See Figure 3 for the sample structure of the experiment). Multi-deviant paradigms have been successfully used in previous studies (Nousak et al., 1996; Sussman et al., 2002; Muller et al., 2005). Only the ERPs from the standard /gu3pa/ tokens were included in the analysis for the standard category (to match the deviant Tone 1 and Tone 2 on vowel /u/). For better control, /gi3pa/, and /gy3pa/ were not included in the analysis although they served as T3 standard stimuli. A stimulus onset asynchrony (SOA) of 900 ms [an average ISI (offset to onset) of 575 ms, range 545–609 ms] was used for the short ISI condition, and of 3,000 ms (average ISI of 2,675 ms, range of 2,645–2,709 ms) for the long ISI condition. The longer ISI condition was considerably longer than most behavioral studies (e.g., 1,500 ms in Werker and Logan, 1985) and ERP studies with speech stimuli because piloting of the ISIs indicated that an ISI longer than 2,500 ms was necessary for sufficient decay of the auditory memory trace to allow observation of language experience effects.

### Behavioral experiments: tone discrimination and identification

A discrimination task was conducted on the same stimuli after the ERP session. The same long and short ISIs were used for the behavioral conditions as for the ERP experiments (an average of 575 and 2,675 ms, respectively). Thirty-three trials including three practice trials were presented. Each trial consisted of a train of five stimuli (four standard followed by a deviant), and participants were asked to judge whether the final stimulus was the same or different from the previous four stimuli. This design was chosen to mimic the ERP design, but it required fewer total trials, and allowed time for a response (up to 4 s between stimulus trains for a response). After the discrimination task, a three-alternative forced choice (3AFC) tone identification task was presented. In this identification task, one stimulus was presented at a time, and participants were asked to press a button (Button 1, 2, or 3) to decide whether the first syllable of the sound was Tone 1, Tone 2, or Tone 3. Six practice trials plus 30 test trials were presented. The behavioral experiments were run after the nonattentive listening ERP experiments to avoid overt learning effect on the ERP responses.

### ERP recording and offline analysis

The electroencephalogram (EEG) was sampled at 500 Hz (filtering bandwidth of 0.1–100 Hz) from 65 scalp sites using Geodesic sensor nets, referenced to the vertex electrode (Cz)^1^. For offline processing, the EEG was refiltered using a finite impulse response (FIR) band-pass filter of 0.3–15 Hz. The phase response of the FIR filter is linear, therefore providing greatest possible accuracy. High pass filter of 0.3 Hz on individual data has negligible distortion to the original data (Rousselet, 2012), while low pass of 15 Hz is adequate for examining MMN given that the MMN has most of its energy in the 2- to 5-Hz frequency band (Picton et al., 2000). The EEG was time-locked to the onset of stimuli and was segmented offline into 1,000 ms epochs including a 200 ms pre-stimulus baseline. Automatic EOG artifact and eye movement artifact correction were applied using Brain Electrical Source Analyses (BESA) (BESA research 5.2, BESA GmbH, Germany). Epochs that exceed the amplitude threshold of 120 μV were excluded, and channels with bad signal throughout the whole recording session were interpolated using the BESA spline interpolation method. After artifact rejection, the majority of participants had over 75% of trials included in the individual average data. The average number (and standard deviations in parentheses) of trials accepted for the three stimulus types are: deviant stimulus /gu1pa/: 177(15), deviant stimulus /gu2pa/: 177(15) and standard stimulus /gu3pa/: 408(35). Data were further re-referenced using average reference.^2^

## Data analyses

### ERP analyses: MMN and LN

**Figure 4:**
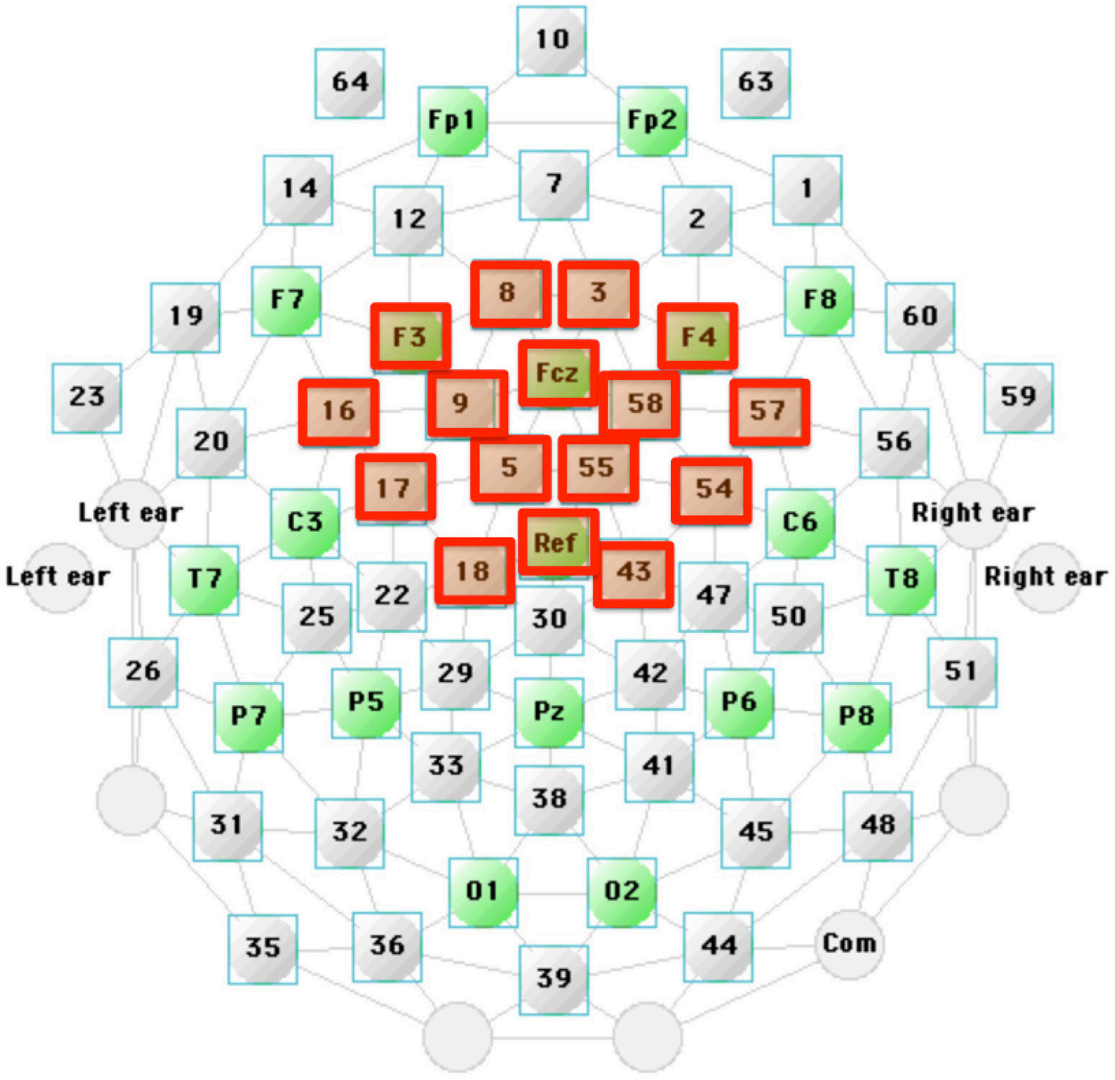
**The Fz composite site was built using the average of the 16 frontocentral sites (highlighted with red squares)**. These 16 sites were selected based on the high positive correlation with Fz.

As found in previous studies and visual examination of the data, MMN was generally largest at Fz. We thus built a model of frontocentral activity from 16 sites as follows. We first calculated the Pearson's correlation coefficients r (related to Global Dissimilarity Index (DISS) by *r* = 1 − DISS^2^/2) (Skrandies, 1990; Murray et al., 2008; Shafer et al., 2011) between each of the 64 channels and Fz for each stimulus condition and each language group. The 16 adjacent fronto-central sites (Geodesic 65-channel net, Electrodes 3, Fz, 5, 8, 9, F3, 16, C3, 18, 43, C4, 55, 57, 58, F4, and Cz) showed high correlations of greater than 0.87 to Fz. Averaging across these sites reduces the contribution of independent noise sources at each electrode site to the signal of interest, and it also reduces the inter-subject variations in the topography of the ERP to speech (Zevin et al., 2010). Thus, the average of these 16 sites was used as the dependent measures (called “composite Fz”) (see Figure 4). The inferior-posterior sites (including the mastoids and sites near P7, P8) were highly negatively correlated with Fz (Pearson *r* values of −0.79 or above), which is consistent with the topography of MMN.

Based on the previous literature (e.g., Kaan et al., 2008; Yu et al., 2014) and visual inspection of the grand average waveforms, we chose the time window of 100–350 ms (five time bins of 50 ms) for the MMN analyses, and 350–600 ms (five time bins of 50 ms) for the LN analyses. The average amplitudes were calculated for each time window for each subject/stimulus condition. We did not use peak amplitude and peak latency because there is sometimes more than one peak in the grand-mean waveforms, so using multiple consecutive time windows is a more objective measure given the features of our data. Furthermore, we would like to examine the interaction between time and amplitude. We and several other groups of researchers have previously used this method of analysis (e.g., Shafer et al., 2004; Hisagi et al., 2010, 2015; Zevin et al., 2010; Lee et al., 2012). Step one analysis was to determine the presence/absence of MMN and LN by comparing the amplitude of the subtraction wave (deviant minus standard) with a hypothetical zero within each time window for each deviant and ISI condition. *P*-values were adjusted for multiple comparisons. Significance levels were reported using adjusted *p*-values.

Step two analyses used the amplitudes of the subtraction waves (deviant minus standard) at the composite Fz as the dependent variable. Four-way mixed model ANOVAs with Language group (English, Mandarin), ISI (short, long) as between-subject variables, and deviant stimulus type (T2, T3) as within-subject variable were undertaken separately for the early time-interval (five intervals from 100 to 350 ms) and the later time intervals (five intervals from 350 to 600 ms) to examine the effect of ISI and language experience on the MMN and LN responses.

### Behavioral analyses

Behavioral discrimination data were analyzed with respect to hit rate and false alarm rates. d-prime sensitivity scores (d' = z (hit) –z (false alarm)) were calculated, and followed by repeated measures ANOVAs. Behavioral identification accuracy was also calculated for each language and tone type, followed by repeated measures ANOVAs.

For all ANOVAs, degrees of freedom were adjusted using Greenhouse-Geisser correction for comparisons with more than one degree of freedom in the numerator and were reported as corrected *p*-values. The uncorrected degrees of freedom, *F*-values, corrected *p*-values and the epsilon (ε) values when applicable were reported.

### Correlation between the ERP and behavioral responses

We also examined the correlations between brain and behavioral discrimination by using Pearson's Product Moment Correlation. Four sets of correlation analyses were performed using the peak amplitude values and peak latency values for MMN and LN as the ERP responses, and the d-prime scores for the discrimination task for the T3-T1 condition and T3-T2 condition as the behavioral responses. The correlations between the MMN and the T3-T1 and T3-T2 discrimination performance and the correlations between the LN responses and the results from the two discrimination tasks were calculated within each participant group.

### Comparison between lexical tones and vowels

We compared the peak amplitudes for the “hard” tone deviant T2 condition and the “hard” vowel deviant /gy3pa/ condition across the language by ISI groups using repeated measures ANOVA. Peak amplitudes were chosen from the average amplitudes of the waveforms across twelve 20 ms time bins between 100 and 340 ms. Language (English, Mandarin), ISI (short, long) and stimulus type (tone, vowel) were the independent variables, and peak MMN amplitude was the dependent variable.

## ERP results

### The presence/absence of MMN and LN

**Figure 5:**
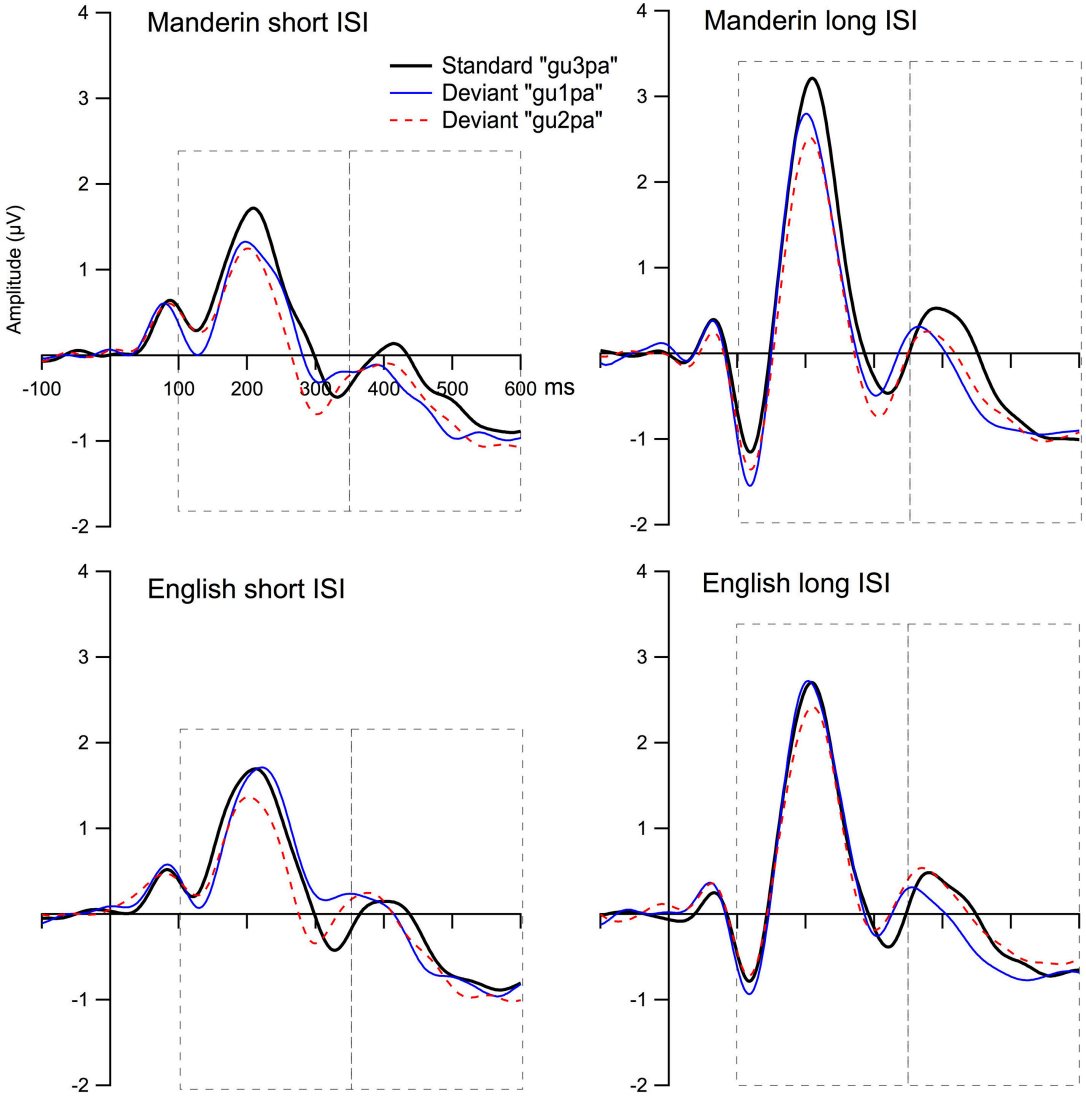
**Grand average ERPs to the standard and deviant stimulus waveforms for the composite Fz**. The top panel shows Mandarin short ISI group T1 and T2 conditions **(left)**, and long ISI group T1 and T2 conditions **(right)**. The bottom panel shows English short ISI group T1 and T2 conditions **(left)**, and long ISI group T1 and T2 conditions **(right)**. The first dash-lined box highlights the time windows of the MMNs (five time intervals between 100 and 350 ms), and the second dash-lined box highlights the time windows of the LNs (five time intervals between 350 and 600 ms).

**Figure 6:**
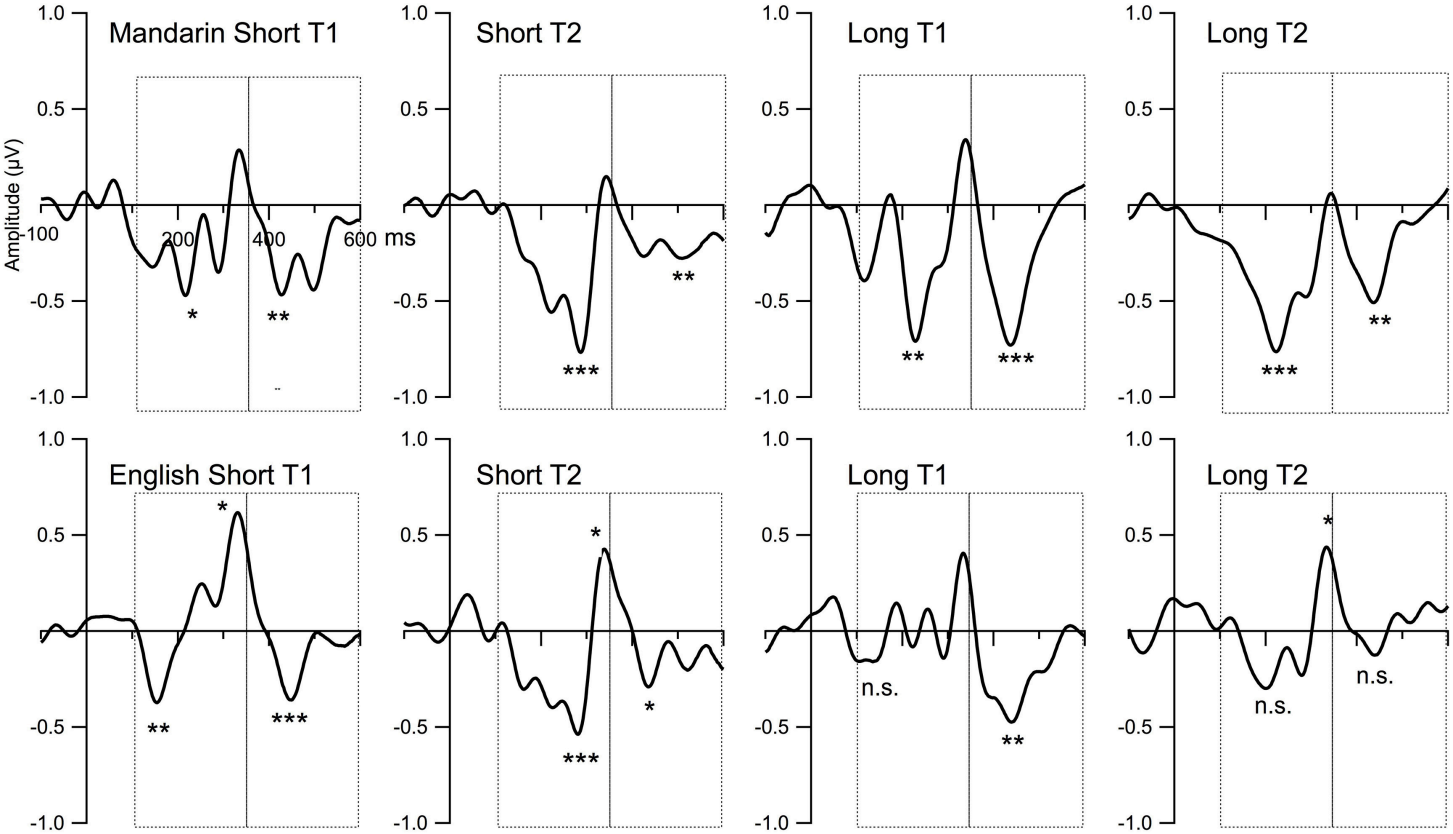
**The MMN (deviant minus standard) for the Mandarin and English groups across two ISI conditions and two deviant tone type conditions**. “Short” stands for a short ISI of approximately 575 ms, and “Long” stands for a long ISI of approximately 2,675 ms. “T1” refers to the deviant tone 1 “gu1pa” condition, and “T2” stands for the deviant tone 2 “gu2pa” condition. The first dash-lined box highlights the time windows of the MMNs (five time intervals between 100 and 350 ms), and the second dash-lined box highlights the time windows of the LNs (five time intervals between 350 and 600 ms). ^*^ For adjusted *p* < 0.05, ^**^ adjusted *p* < 0.01, ^***^ adjusted *p* < 0.001, and n.s. means not significant.

Figure 5 displays the grand mean ERPs to the standard and deviant stimulus waveforms for the composite Fz, and Figure 6 shows the grand mean subtraction waveforms for the composite Fz for the two language groups under four ISI (2) by deviant tone type (2) conditions.

Table 3 shows the presence/absence of MMN for all participant groups using adjusted *p*-values. For Tone 1-Tone 3 (T1-T3) contrast, no MMN was present for the English Long ISI (EL) group, and MMN was present only between 150 and 200 ms for the English Short ISI (ES) group; for the Mandarin listeners under T1-T3 contrast, MMN was present in three of the five time windows for the Mandarin Long ISI (ML) group, and also present between 150 and 200 ms for the Mandarin Short ISI (MS) group. For Tone 2-Tone 3 (T2-T3) contrast, again, no significant MMN was present for the EL group, while MMN was significant between 200 and 350 ms for the ES group, and between 200-300 ms for ML group, and between 150 and 350 ms for the MS group. Note that from Figure 6, it appears that there might be a difference for T2/T3 contrast. However, statistically, that difference did not reach significance level due to large variance. See Table 3, the largest average amplitude for T2/T3 condition is −0.33 μV, but the standard deviation is 0.52 μV.

**Table 3:** **MMN amplitudes at Composite Fz (“EL” = English long ISI, “ES” = English short ISI, “ML” = Mandarin long ISI, “MS” = Mandarin short ISI)**.

			**T3-T1**					**T3-T2**		
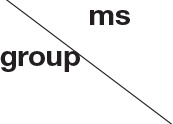	**100** **−150**	**150** **−200**	**200** **−250**	**250** **−300**	**300** **−350**	**100** **−150**	**150** **−200**	**200** **−250**	**250** **−300**	**300** **−350**
EL	−0.13	−0.16	0.09	0.11	−0.11	0.05	−0.05	−0.33	−0.12	−0.04
*(SD)*	*0.26*	*0.51*	*0.47*	*0.52*	*0.64*	*0.31*	*0.37*	*0.52*	*0.48*	*0.41*
ES	0.03	−0.41^b^	−0.15	0.11	0.15	−0.06	−0.27	−0.42^c^	−0.48^b^	−0.39^a^
*(SD)*	*0.40*	*0.41*	*0.39*	*0.58*	*0.47*	*0.43*	*0.39*	*0.43*	*0.46*	*0.47*
ML	−0.33^a^	−0.17	−0.34^b^	−0.49^a^	−0.18	−0.19	−0.34^a^	−0.64^b^	−0.57^b^	−0.46
*(SD)*	*0.46*	*0.39*	*0.43*	*0.67*	*0.56*	*0.54*	*0.47*	*0.62*	*0.59*	*0.74*
MS	−0.17	−0.25^a^	−0.33	−0.10	−0.22	0.01	−0.22	−0.40^b^	−0.42^b^	−0.57^c^
*(SD)*	*0.40*	*0.39*	*0.70*	*0.45*	*0.48*	*0.35*	*0.28*	*0.39*	*0.39*	*0.40*

Superscript ^a^ means adjusted p < 0.05;

Superscript ^b^ means adjusted p < 0.01;

*Superscript ^c^ means adjusted p < 0.001*.

Table 4 shows the presence/absence of LN for all participant groups. For the T1-T3 condition, LN was significant in the ms group between 500 and 550 ms, and LN was present in the rest three groups between 450 ms and 500 ms. For the T2-T3 condition, no LN for the EL group, and the LN was significant between 500 and 550 ms in the ES group, between 450 and 500 ms for the ML group and 500–600 ms for the ms group.

**Table 4:** **LN amplitudes at Fz composite (“EL” = English long ISI, “ES” = English short ISI, “ML” = Mandarin long ISI, “MS” = Mandarin short ISI)**.

**Tone 3-Tone1**						**Tone 3- Tone 2**				
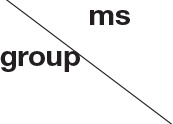	**350** **−400**	**400** **−450**	**450** **−500**	**500** **−550**	**550** **−600**	**350** **−400**	**400** **−450**	**450** **−500**	**500** **−550**	**550** **−600**
EL	0.19	−0.46	−0.56^a^	−0.33	−0.03	0.29^a^(+)	−0.07	−0.18	0.06	0.13
*(SD)*	*0.52*	*0.79*	*0.69*	*0.72*	*0.48*	*0.41*	*0.26*	*0.47*	*0.48*	*0.30*
ES	0.34	−0.13	−0.38^b^	−0.17	−0.17	0.27^a^(+)	−0.14	−0.23	−0.23^a^	−0.18
*(SD)*	*0.57*	*0.4*	*0.35*	*0.44*	*0.40*	*0.34*	*0.49*	*0.42*	*0.37*	*0.34*
ML	0.19	−0.46	−0.67^c^	−0.20	−0.04	0	−0.41	−0.53^a^	−0.15	−0.10
*(SD)*	*0.63*	*0.68*	*0.51*	*0.3*	*0.42*	*0.7*	*0.79*	*0.61*	*0.37*	*0.31*
MS	0.19	−0.2	−0.33	−0.45^a^	−0.08	0.17	−0.16	−0.18	−0.27^b^	−0.21^b^
*(SD)*	*0.63*	*0.56*	*0.61*	*0.48*	*0.27*	*0.64*	*0.57*	*0.44*	*0.36*	*0.35*

Superscript ^a^ means adjusted p < 0.05;

Superscript ^b^ means adjusted p < 0.01;

*Superscript ^c^ means adjusted p < 0.001*.

### The effect of ISI and deviant tone type conditions for MMN (100–350 ms)

Repeated measures ANOVA revealed a main effect of language [*F*_(1, 59)_ = 6.177, *p* = 0.01], main effect of deviant tone type [*F*_(1, 59)_ = 9.590, *p* = 0.002], and interactions between tone type and time [*F*_(4, 236)_ = 14.1, *p* < 0.0001, ε = 0.87] and tone type by ISI by time [*F*_(4, 236)_ = 5.544, *p* < 0.001, ε = 0.87]. *Post-hoc* tests for the main effects showed that the two Mandarin groups had larger MMN amplitudes than the two English groups. The T2 deviant elicited a larger MMN amplitude than the T1 deviant for all groups. *Post-hoc* tests following up the time by deviant tone-type interaction revealed that T2 was more negative than T1 between 200 and 350 ms. To follow the three-way interactions, step-down analyses using ISI and time for each language and tone type respectively were performed. MMN is the largest for the T1 condition between 200 and 250 ms for English listeners. In the T2 condition for English listeners, there was a main effect of ISI, specifically, the MMN is larger in the short ISI condition than in the long ISI condition [*F*_(1, 29)_ = 5.22, *p* = 0.03], and there was also a main effect of time, with the larger MMN amplitudes in the 200–300 time window than the other time windows [*F*_(4, 16)_ = 4.09, *p* = 0.003, ε = 0.75]. No ISI or time effect was observed in the Mandarin groups for T1, and no ISI effect in the Mandarin group for T2, either. The only significant effect was time [*F*_(4, 120)_ = 10.9, *p* < 0.001, ε = 0.84]. *Post-hoc* tests revealed that the MMN for T2 was larger in the three later time windows between 200 and 350 ms than in the first two time intervals. In summary, the Mandarin groups have larger MMNs than the English groups independent of deviant conditions; for the English group, MMNs were larger the short ISI conditions, especially for the T2 deviant condition.

### The effect of ISI and deviant tone type conditions for LN (350–600 ms)

The results from the ANOVA using the subtraction waves showed significant interactions of ISI by time [*F*_(4, 236)_ = 4.29, *p* = 0.002, ε = 0.65], and tone type by time [*F*_(4, 236)_ = 4.89, *p* = 0.001, ε = 0.78]. *Post-hoc* tests did not locate the specific difference for the ISI by time interaction, however, it did show that for the tone type by time interaction, the LN amplitude for T1 was larger than for T2 between 450 and 500 ms. No main effect or interaction involved the language variable for T1 under either short or long ISI conditions, but there was a main effect of language for T2 [*F*_(1, 29)_ = 5.156, *p* = 0.03] with Mandarin listeners showing larger LN amplitude. Step-down analyses were performed to examine the effect of ISI on the LN responses within each language group and deviant tone type. For the Mandarin group T1-T3 condition, there is a significant interaction of time and ISI [*F*_(4, 120)_ = 3.46, *p* = 0.02, ε = 0.73]. *Post-hoc* tests did not find any specific significance although it appears that the LN amplitude is larger for the ML group than the MS group for T1-T3 condition between 400 and 500 ms. No other significant interactions involving ISI for either the Mandarin or English groups were found. That is, the LN amplitude is in general larger for T1 than for T2 between 450-500 ms, and the Mandarin group showed larger LN for T2 condition than the English group. Longer ISI generated larger LN for the Mandarin groups under T1-T3 condition.

### Behavioral discrimination and identification

Table 5 displays the d' scores reflecting discrimination accuracy. Both Mandarin and English listeners performed the discrimination task with greater than chance level accuracy under the short and long ISI conditions. There was a main effect of language group [*F*_(1, 59)_ = 10.27, *p* < 0.01], and a main effect of lexical tone type [*F*_(1, 59)_ = 47.3, *p* < 0.0001]. Mandarin listeners showed higher accuracy scores than English listeners, and the tone 1- tone 3 contrast (T3-T1) condition showed higher accuracy than the tone 3 - tone 2 contrast (T3-T2) condition. An ISI by language group interaction was also significant [*F*_(1, 59)_ = 4.204, *p* = 0.04]. Tukey's *post-hoc* tests show that the English group had lower accuracy than the Mandarin group in the long ISI condition. *Post-hoc* on the language by tone type interaction shows that the Mandarin group discriminated T3-T1 and T3-T2 with similar accuracy, but higher performance for T3-T1 contrast than T3-T2 contrast was observed in the English participants.

**Table 5:** **Behavioral discrimination accuracy d-prime sensitivity scores [d' = z (hit) –z (false alarm)] for each language, interstimulus (ISI) and tone contrast conditions (T3/T1 means Tone 3 vs. Tone 1; T3/T2 means Tone 3 vs. Tone 2)**.

	**T3/T1**		**T3/T2**	
	**Average**	***SD***	**Average**	***SD***
Mandarin-Short ISI	4.64	1.88	3.22	2.53
Mandarin-Long ISI	5.12	1.14	4.33	1.53
English-Short ISI	4.15	1.94	1.88	1.91
English-Long ISI	3.81	3.17	0.79	2.23

There was an expected large difference in the response patterns of the two language groups in terms of behavioral identification results (See Table 6). The Mandarin listeners identified all three tone types well-above chance (>33%) while the English listeners were at or below chance level for all three tone types. Mixed measure ANOVAs using language as the between-subject variable and tone type as the within-subject variable revealed a language main effect [*F*_(1, 61)_ = 68.6, *p* < 0.001], a tone type main effect [*F*_(2, 122)_ = 8.48, *p* < 0.001, ε = 0.96] and an interaction of tone type by language group [*F*_(2, 122)_ = 5.91, *p* = 0.004, ε = 0.96]. *Post-hoc* tests showed that T2 was identified with the highest accuracy, and this effect was driven by the English listeners. The Mandarin listeners identified the three tones equally well.

**Table 6:** **Behavioral identification accuracy (1 = 100% accuracy, and 0.33 is at chance accuracy)**.

	**T1: Mean (*SD*)**	**T2: Mean(*SD*)**	**T3: Mean(*SD*)**
Eng_Long	0.23 (0.29)	0.57 (0.22)	0.16 (0.20)
Eng_Short	0.29 (0.34)	0.51 (0.28)	0.49 (0.33)
Mand_Long	0.73 (0.39)	0.83 (0.24)	0.85 (0.26)
Mand_Short	0.82 (0.30)	0.82 (0.18)	0.87 (0.18)

### Correlation between the brain responses and behavioral discrimination

Table 7 shows that there was no significant correlation between the ERP responses and the behavior for any language/ISI group under either stimulus condition for either MMN or LN peak amplitude or peak latency.

**Table 7:** **Correlations between ERP and behavioral responses**.

	**T1-T3**		**T2-T3**	
	**MMN-DISC**	**LN-DISC**	**MMN-DISC**	**LN-DISC**
**ERP amplitude and DISC**				
English Short ISI	0.23	−0.22	0.07	−0.11
English Long ISI	0.3	0.17	−0.24	0.12
Mandarin Short ISI	0.33	0.26	0.06	−0.17
Mandarin Long ISI	−0.12	−0.35	0.42	0.23
**ERP latency and DISC**				
English Short ISI	0.04	0.05	−0.42	−0.12
English Long ISI	0.32	−0.13	0.01	−0.14
Mandarin Short ISI	0.03	−0.08	0.07	<0.01
Mandarin Long ISI	−0.04	−0.43	0.23	−0.21

*The correlation between each of the two ERP components (MMN and LN) and behavioral discrimination responses was calculated for both T1-T3 contrast and T2-T3 contrast. Threshold for significant correlation (p < 0.05) is |r| > 0.53. No significant correlations were found*.

### Comparison between lexical tone and vowel conditions for peak MMN amplitude

**Figure 7:**
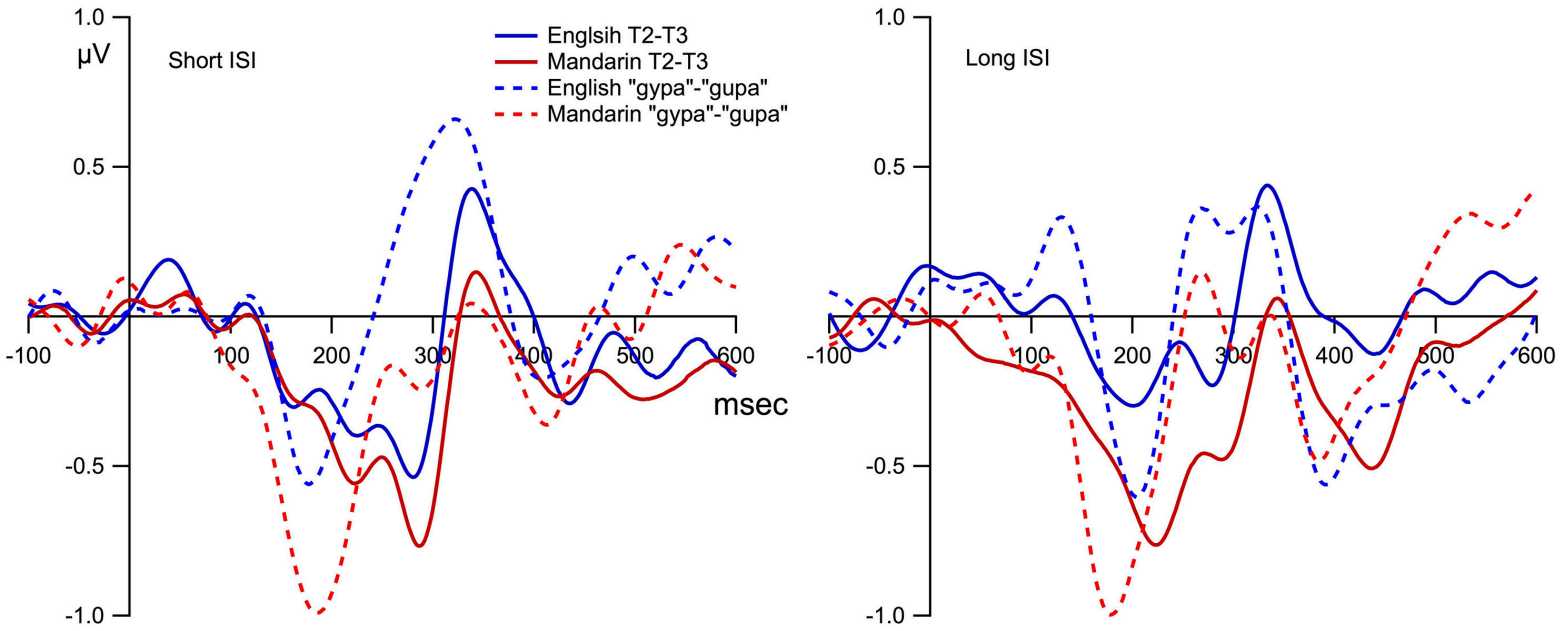
**Comparison of the mismatch responses for deviant Tone 2 condition and deviant “gypa” condition**.

A vowel mismatch condition was included with the purpose of increasing variability and to allow some perspective on the latency and amplitude of the vowel MMN (which was earlier) compared to lexical tone MMN. Thus, we have not included a full analysis of the vowel data. However, for comparison purposes, we include one analysis comparing the lexical tones to the vowels. Figure 7 displays the subtraction waveforms for deviant T2 condition and deviant /gy3pa/ condition for all language by ISI groups. Repeated measures ANOVA revealed a significant main effect of language [*F*_(1, 59)_ = 9.63, *p* = 0.003] with the Mandarin listeners showing overall larger MMN amplitude, and a main effect of stimulus type [*F*_(1, 59)_ = 4.26, *p* = 0.04] with the vowel deviant condition (/gypa/) eliciting overall larger MMN than the tone deviant condition (/gu2pa/). No other main effect or interaction reached significance.

## Discussion

The current study was designed to extend our understanding of the neural correlates of lexical tone processing. The time period (ISI) that the acoustic-phonetic information needed to be retained in sensory memory for discrimination was manipulated to allow us to evaluate long-term memory support for lexical tone processing. The main finding was that MMN amplitude was of similar amplitude in the short and long ISI conditions for Mandarin listeners. In contrast, the English groups showed diminished or absent mismatch responses for the long compared to the short ISI condition. In particular, the Mandarin and English listeners did not show a difference in MMN amplitude for the short ISI condition. This pattern of findings better supports the explanation that listeners were relying on long-term memory representations to update sensory memory, and that English speakers' long term representation for tone information was inadequate to support discrimination. English listeners' long-term representations of F0 may encode information that is necessary for lexical stress or for sentence level prosody, but these representations would likely weigh F0 information in a manner that is insufficient to support lexical tone perception. Below we discuss these findings in greater detail in relation to the current literature on neural plasticity of lexical tone processing and the role of ISI on speech processing.

### ISI and behavioral responses

The behavioral findings of a larger discrimination accuracy difference between the Mandarin and English groups under the long ISI conditions were consistent with previous studies, in that they revealed reliance on the phonemic level of processing at longer ISIs (Pisoni, 1973; Werker and Tees, 1984; Werker and Logan, 1985; Burnham et al., 1996). We extended the current behavioral literature by comparing the acoustically more similar T2-T3 contrast vs. the acoustically more distinct T1-T3 contrast across two ISI conditions, which allowed us to examine the interaction between acoustic distance and sensory memory trace decay. We found that English listeners can use phonetic information to discriminate the tones. However, they cannot easily discriminate the tones under the long ISI conditions because the rapid decay of acoustic/phonetic information in the longer ISI condition leads to greater reliance on phonemic processing, and the tone contrasts in this study are not phonemic for the English listeners.

Our behavioral discrimination experiments differed from the AX paradigms used by previous studies (Pisoni, 1973; Werker and Logan, 1985; Burnham et al., 1996). We adopted a modified version from the passive ERP oddball paradigm (A_1_A_1_A_2_A_1_X or A_1_A_2_A_1_A_1_X) for our behavioral task to allow more direct comparison with the neurophysiological responses. Another difference is that our long ISI condition was considerably longer than that used in these previous studies comparing discrimination. Most studies used 500 vs. 1,500 ms whereas we used 575 vs. 2,675 ms. As discussed in the methods section, this longer ISI was selected because piloting using a 1,500 ms ISI revealed little or no decline in the MMN amplitude compared to a 500 ms ISI. The need for longer ISI to observe the decline of MMN amplitude may indicate a dissociation between behavioral and neurophysiological measures under certain conditions. Alternatively, it is possible that the difference in the physical properties of tones compared to other phonemic categories (such as consonants) was responsible for sensory memory decay difference. Further studies examining differences in non-speech stimuli that have similar physical properties to speech will be necessary to determine which explanation is better.

Lang et al. (1990) was the first study that reported MMN response to small stimulus contrast could predict behavioral discrimination accuracy. Recently, Koerner et al. (2016) found that MMN latency but not MMN amplitude predicted phoneme detection accuracy. However, we did not find any correlation between behavioral discrimination accuracy and MMN responses or discrimination accuracy and LN responses. This result is consistent with the findings of Chen and Sussman (2013). The lack of MMN and behavioral discrimination correlations have also been reported in other studies (e.g., Shafer et al., 2004; Horváth et al., 2008). Thus it appears that the relationship is not linear, but rather categorical (that is, bilingual experience leads to better behavior and larger MMN in the long ISI condition, but in a non-linear fashion).

### ISI as a probe for speech sound representation

We had predicted that native-language experience would allow robust brain discriminative responses for lexical tone in the face of decay of the immediate memory trace. Our findings are consistent with this claim. The Mandarin listeners showed robust MMN in the long ISI conditions while the English listeners showed no MMN under this condition for both deviant tone types. This finding better supports an explanation of the memory trace for speech information being supported by long-term memory representations. The alternative explanation was that experience somehow leads to more salient representation of relevant cues in sensory memory, as we have suggested previously (e.g., Hisagi et al., 2010). However, it is possible that because tone differences are more robust than some segmental differences (for example formant differences in vowels) tone discrimination was too easy at the short ISI, and thus did not allow us to see language group differences at this short ISI.

Past research found that a longer ISI leads to less prominent or absent MMN (for pure tone, Pekkonen et al., 1996; Gomes et al., 1999; Barry et al., 2008; Grossheinrich et al., 2010; for speech, Čeponiene et al., 1999). These studies suggest that the duration of the auditory sensory memory store is reflected in the MMN. The majority of these studies used pure auditory tones of contrasting frequencies as stimuli, and thus, the results of ISI manipulations primarily reflect auditory sensory memory decay because it is less likely that long-term representations of pure tones with relatively small frequency differences are stored without relevant training (Hedger et al., 2013). Among the few ERP experiments using speech contrasts, only one ERP study used an ISI longer than 1.5 s. Čeponiene and colleagues used a consonant contrast in bisyllabic nonwords (/baga/ and /baka/) and found that children with good phonological memory showed a smaller MMN for a long (2 s) ISI compared to a short (350 ms) ISI, and no MMN was seen in children with poor phonological memory (Čeponiene et al., 1999). Based on these findings and our own results, it is clear that when the ISI is long, the amplitude of MMN is particularly sensitive to the language status of the listener. In two other related studies (which were not designed to directly examine ISI differences), typically developing children showed an earlier MMN to a long 250-ms vowel contrast ([ε] vs. [I]) presented using a short ISI of 350 ms compared to a short 50 ms-version of the same contrasts presented with a longer ISI of 550 ms; furthermore, many of the children with specific language impairment (SLI) did not exhibit a robust MMN to the short vowel/long ISI condition, but almost all of the children with SLI in the second study showed robust MMN to the long vowel/short ISI condition (Shafer et al., 2005; Datta et al., 2010). Thus, the stimulus duration and/or ISI could have led to this pattern of findings.

Our study adds to the literature on sensory memory decay for speech processing and shows that experience with a specific speech sound influences the apparent time course of sensory memory decay. In addition, the current study also showed that the degree of stimulus difference influences the apparent decay rate. We say “apparent” time course of decay because the time course of decay, *per se*, is not changing. A very large acoustic difference between two stimuli (e.g., 1,000–1,100) may give the appearance of longer maintenance of information in memory compared to a smaller acoustic difference. The current study suggests that what was considered to be a large difference is dependent on both acoustic and experiential factors. In the future, the use of multiple acoustic differences and multiple ISI measures will provide more specific information regarding the nature and time-course of sensory memory decay and how this is influenced by acoustic, phonetic and phonological factors.

### Interaction between ISI and acoustic salience

Our finding that the two language groups differ most under the long ISI conditions for the more difficult T3/T2 contrast corroborates and extends the previous behavioral literature (Gandour and Harshman, 1978; Wang et al., 1999). Our behavioral data showed that there was a striking difference in the English groups for discriminating both tone contrasts. The English groups were much poorer in discriminating T3/T2 than T3/T1, while Mandarin listeners showed discrimination accuracy of over 90% for both T3/T2 and T3/T1. According to Burnham (1986), and expanded by Strange (2011), contrast salience depends upon the size of acoustic change, as well as listener's experience with the phonetic contrast. Behavioral performance in the present study suggests that the T3/T2 might be a “fragile” contrast for English listeners, but not necessarily so for Mandarin listeners. However, this result is at odds with the findings of the Chandrasekaran et al. (2007b) study. In Chandrasekaran et al. (2007b), the two language groups differed under the “easy” T3/T1 condition, but not the “hard” T3/T2 condition. In their study, MMNs to T1/T3 and T2/T3 for English listeners were equally small and comparable to the Chinese T3/T2 condition. One explanation for the difference between their study and ours is that Chandrasekaran and colleagues used a relatively short ISI similar to our short ISI condition. Our ERP results from the short ISI condition of about 550 ms (SOA = 900 ms) show that there was no language group difference at fronto-central sites. It is possible that the larger MMN for the fronto-central measure under T3/T1 than T3/T2 at the fronto-central sites in Chandrasekaran et al. (2007b) was because they used a linked mastoid reference. Also, it is important to keep in mind that language experience differences were found in the later time interval in our study and that it is possible that this “LN” reflects the change detection process for the contour shape, and really is simply a late MMN. Recall that a late effect can also be seen in Chandrasekaran et al. (2007b) Figure 1. This will be discussed further in the next sections. MMN was present under the short ISI condition for a small acoustic contrast, but absent in the long ISI condition for both large and small acoustic contrast indicating there is at least an interaction between acoustic salience and sensory memory.

### LN and lexical tone processing

The results of our study fill the important gaps in the literature on the later stage of auditory sensory processing of lexical tone because the LN component has rarely been examined for lexical tone processing (e.g., Kaan et al., 2007). There is only one study other than ours that has examined the LN responses to lexical tone deviance (Kaan et al., 2007). In Kaan et al. (2007), a high-rising tone/mid tone contrast did not generate an MMN in Thai, Chinese or English listeners, but elicited a late negativity. In contrast, a low-tone deviant generated MMN, but no LN. Examining the tone contour of stimuli in Kaan et al. (2007), it appears that the significant acoustic difference between the standard and the high-rising deviant occurs almost 300 ms after stimulus onset; thus it is possible that what the LN in Kaan et al study for the high-rising deviant condition is actually the MMN to the contour change.

Comparing our results to the general literature on LN, our findings that the overall larger LN in the Mandarin group than in the English group and larger LN for the acoustically more distinct contrast (T3-T1 condition) than the less distinct contrast (T3-T2 condition) diverge from those of some previous studies in which a larger LN is sometimes seen in the less experienced group (e.g., family of specific language impairment or SLI in Addis et al., 2010; late bilingual learners in Ortiz-Mantilla et al., 2010) or impaired listeners (e.g., children with SLI in Shafer et al., 2005). There are also a few studies showing reduced LN in children with dyslexia (Neuhoff et al., 2012; Halliday et al., 2014). Furthermore, our results on LN were not clear-cut. The two conditions in which the LN was significantly diminished are the short ISI T2 deviant condition in the Mandarin group and the long ISI T2 deviant condition for the English group. We propose that the underlying reasons for diminished LN in Mandarin listeners is different than the reason for a diminished LN in English listeners. The lack of LN in the short ISI condition for the Mandarin listeners may suggest the automaticity of the process, while the lack of LN in the long ISI condition for English listeners may indicate insufficient support for further processing of the stimulus contrast. The interpretation of the functional features of LN is far from conclusive. Several studies proposed that LN might be attributed to an increase in involuntary shifting/reorienting of attentional mechanisms (speech stimuli: Shestakova et al., 2003; Auditory tone contrast: (Schröger and Wolff, 1998); auditory tone contrast: Ortiz-Mantilla et al., 2010). Researchers such as Korpilahti et al. (2001) proposed that LN can be considered “the second MMN,” and it reflects further processing of the stimuli in the semantic domain. Along the similar line, Shafer and colleagues suggested that the LN for speech contrast indicates further processing that is independent of phonological representation (Shafer et al., 2005).

As discussed in Shestakova et al. (2003), we agree that the function of LN may differ across various tasks and participant characteristics. Specifically in our study, it is possible that the mechanism indexed by an LN in a long ISI condition differs from those indexed by LN in a short ISI listening context, and that the long ISI context automatically recruited more higher-level cognitive resources. An additional possibility is that the LN for the Mandarin listeners is the MMN to the tone contour, which necessarily is later in time because the difference cannot be computed until the end of the syllable. Even if this is the case, however, the co-occurance of enhanced and reduced negativity needs to be further explained. We also found positivity in the early portion of this 300-500 ms interval for the short ISI conditions. It is possible that this positivity is a P3a orienting response that partially overlaps with the LN (Gumenyuk et al., 2005). In this case the apparent enhancement of the LN at long ISIs may be due to absence of the overlapping P3a.

Furthermore, the current study used bisyllabic stimuli, and the second syllable is always /pa1/ with Tone 1 (a high level tone). However, due to coarticulation effects, the F0 contour for /pa1/ is affected by the tone status of the preceding syllable and had the highest values when preceded by T1 context (e.g., gu1pa), the lowest values for T3 context (e.g., gu3pa), and the intermediate values when preceded by T2 (e.g., gu2pa). It is possible that an F0 difference also contributes to the negativity we observed in the 350-550 ms time window. The pitch difference on the second syllable /pa1/ is a within-category distinction for both Mandarin and English listeners; therefore, it is more likely to generate similar discrimination responses from the two language groups.

In summary, our results add to an increasingly complicated picture regarding the functional nature of the LN. As suggested in behavioral findings, the LN to lexical tone contrasts may differ from consonant and vowel processing. Depending on the specific tone contrast, it may reflect a later MMN-type process to the contour shape, or different levels of automaticity in processing the stimulus contrast. Clearly, further studies are needed to expand our understanding of LN for lexical tone processing.

### The use of more complex stimuli

In this study we used several strategies, in addition to increasing ISI, to minimize the possibility of using acoustic-phonetic processing alone and to minimize the influence of semantic knowledge. First, the use of nonsense stimuli was intended to minimize the influence of lexical knowledge on listeners' performance given that lexical knowledge/ biases can have an effect on response patterns for both L1 and L2 learners (Best and Tyler, 2007; Strange, 2011). Second, we used multiple oddballs and more than one token per stimulus type, to greatly reduce the number of repeating identical tokens, which would force greater reliance on more abstract patterns; Third, we used natural speech and bisyllabic nonwords to create an ecologically more-valid task and a context that is more likely to preclude reliance on acoustic/phonetic cues alone. Such a paradigm taps into phonemic processing that is based on one's long-term language experience to a greater extent than the use of a single oddball deviant token and a short ISI. However, implementing natural and multi-token bisyllabic stimuli results in less precise control of F0 contour. In addition, it is difficult to construct within-category contrast pairs that share the same acoustic difference as across category pairs. Studies using monosyllabic stimuli such as Xi et al. (2010) enhanced our knowledge about the role of language experience on the neural mechanism of lexical tone processing. Our study adds to the literature by focusing on examining how the strength of the auditory sensory memory as modulated by ISI interacts with lexical tone processing. Both approaches provide valuable information. It will be interesting in a future study to examine how ISI modulates the MMN and LN responses for within-category vs. between-category F0 contrast in both Mandarin and English groups.

### MMN for lexical tone vs. vowel deviants

Even though the main goal of this experiment was to examine lexical tone processing, it was useful to compare the MMN of the difficult lexical tone contrasts to the more-difficult vowel contrast. We found that the MMN for vowel deviants (/gy3pa/) was larger than lexical tone deviant /gu2pa/. Both /gy3pa/-/gu3pa/ and /gu2pa/-/gu3pa/ contrasts are not phonological for monolingual English listeners; therefore larger MMN for native Mandarin speakers was consistent with the previous literature. The larger MMN to vowels than to lexical tone deviants regardless of ISI appears to suggest that for native lexical tone language speakers, the rate of sensory memory decay for lexical tone differs from that for vowels. This is new neurophysiological evidence supporting some behavioral literature on the differences between vowel and lexical tone processing. For example, Wiener and Turnbull (2016) recently reported that it was easier for native Mandarin speakers to modify the lexical tone than the vowel portion of a syllable. Their explanation of this finding is that listeners rely more on the vowel identity than tone identity in lexical processing. In another study, Cutler and Chen (1997) found evidence suggesting that processing of lexical tone distinctions is relatively slower than found for segmental distinctions. It is possible that the larger and earlier MMN to the vowel than to the lexical tone contrasts reflects this relative importance and faster processing. However, it also should be recognized that it is not clear how to equate vowel difference and lexical tone difference. A different vowel contrast for which there is less spectral difference (e.g., /I/ in “pit” vs. /E/ in “pet”) might result in a smaller MMN that seen for the vowel contrast selected here (e.g., Hisagi et al., 2015). Further studies will be needed to understand how vowels and lexical tones are processed at the cortical and behavioral levels.

## Conclusions

This is the first study that examined the neural mechanisms involved in the decay of echoic sensory memory for phonemic, lexical tone contrast. Our study illustrates that the sensory memory trace elicited by the suprasegmental F0 contrast decays within 3 s to an extent that will not support lexical-tone discrimination, without the support of language-specific, long-term memory representations. The results from this study also demonstrated that the phonological information of Mandarin lexical tone have distinct impacts on the later stage of neurophysiological processing as revealed by LNs in the Mandarin and English listeners. Further studies are needed to better understand how stimulus, participant and task modulate the later stage of speech processing as measured by LN. We chose a between-subject design to minimize learning and fatigue effects and avoid order effects that would result from a within-subject design, but this also increased the variance.

## Ethics statement

The study was approved by the human subject research institutional review board at the Graduate Center, City University of New York, and was conducted in compliance with the Declaration of Helsinki. Each potential participant was given informed consent prior to his or her participation of the study. The informed consent include the title of the study, the location of the study, and the procedure (steps, duration, and expected behavior) of the study, the benefits and potential risks of the study. Each potential participant was informed about the confidentiality procedures that the researchers will take. He or she has the full liberty to withdraw from the study at anytime during the experiment without any penalty. Only those who read the informed consent that was approved by the Institute of Review Board of the Graduate Center, City University of New York, and signed the informed consent were recruited to the study. This study did not recruit any vulnerable populations.

## Author contributions

Methodology: YY and VLS; Data curation: YY and VLS; Data analysis: YY and VLS; Result validation: YY, VS, and ESS; Writing original draft: YY; Writing-review and editing: VLS, ESS, and YY.

## Funding

This project was funded by Rees Dissertation Fellowship at the Graduate Center, City University of New York. This project was also funded by the National Institutes of Health (#HD46193 to VLS, and #DC004263 to ESS).

### Conflict of interest statement

The authors declare that the research was conducted in the absence of any commercial or financial relationships that could be construed as a potential conflict of interest.

## Footnotes

^1^The Electric Geodesics, Inc. *EEG system net station* (4.1.2 ed.) EGI, Eugene, Oregon, USA.

^2^BESA research 5.2, Brain Electrical Source Analyses, BESA GmbH, Germany. doi: 10.1017/S0142716400007542
